# Al_1−x_Sc_x_N Thin Films at High Temperatures: Sc-Dependent Instability and Anomalous Thermal Expansion

**DOI:** 10.3390/mi13081282

**Published:** 2022-08-08

**Authors:** Niklas Wolff, Md Redwanul Islam, Lutz Kirste, Simon Fichtner, Fabian Lofink, Agnė Žukauskaitė, Lorenz Kienle

**Affiliations:** 1 Department of Material Science, Faculty of Engineering, Kiel University, Kaiserstr. 2, D-24143 Kiel, Germany; 2 Fraunhofer Institute for Applied Solid State Physics, IAF, Tullastr. 72, D-79108 Freiburg, Germany; 3 Fraunhofer Institute for Silicon Technology ISIT, Fraunhoferstr. 1, D-25524 Itzehoe, Germany

**Keywords:** aluminum scandium nitride, thermal stability, structure analysis, X-ray diffraction, ferroelectrics

## Abstract

Ferroelectric thin films of wurtzite-type aluminum scandium nitride (Al_1−x_Sc_x_N) are promising candidates for non-volatile memory applications and high-temperature sensors due to their outstanding functional and thermal stability exceeding most other ferroelectric thin film materials. In this work, the thermal expansion along with the temperature stability and its interrelated effects have been investigated for Al_1−x_Sc_x_N thin films on sapphire Al_2_O_3_(0001) with Sc concentrations *x* (*x* = 0, 0.09, 0.23, 0.32, 0.40) using in situ X-ray diffraction analyses up to 1100 °C. The selected Al_1−x_Sc_x_N thin films were grown with epitaxial and fiber textured microstructures of high crystal quality, dependent on the choice of growth template, e.g., epitaxial on Al_2_O_3_(0001) and fiber texture on Mo(110)/AlN(0001)/Si(100). The presented studies expose an anomalous regime of thermal expansion at high temperatures >~600 °C, which is described as an isotropic expansion of *a* and *c* lattice parameters during annealing. The collected high-temperature data suggest differentiation of the observed thermal expansion behavior into defect-coupled intrinsic and oxygen-impurity-coupled extrinsic contributions. In our hypothesis, intrinsic effects are denoted to the thermal activation, migration and curing of defect structures in the material, whereas extrinsic effects describe the interaction of available oxygen species with these activated defect structures. Their interaction is the dominant process at high temperatures >800 °C resulting in the stabilization of larger modifications of the unit cell parameters than under exclusion of oxygen. The described phenomena are relevant for manufacturing and operation of new Al_1−x_Sc_x_N-based devices, e.g., in the fields of high-temperature resistant memory or power electronic applications.

## 1. Introduction

Thin films of scandium-substituted aluminum nitride (Al_1−x_Sc_x_N or ‘AlScN’) are a pioneering new class of ferroelectric materials with wurtzite-type structure and unidirectional polarization reversal resulting in square-like hysteresis loops [1,2,3,4,5]. Their ultra-stable remnant polarization and high coercive fields promise ferroelectric field-effect transistor-based non-volatile memory devices maintaining large memory windows combined with high access speed, high endurance and low energy consumption [6]. In this respect, the integration of AlScN on Si has been demonstrated recently for ferroelectric field-effect transistors as well as GaN technology-based structures, e.g., for high electron mobility transistors [7,8,9,10] with integrated memory functions. Further, thin films of AlScN are potential candidates for high-temperature actuation and sensing applications in harsh environments (T > 500 °C) or for high-temperature non-volatile memory due to remarkable temperature stability of the wurtzite-type structure and its piezo/ferroelectric properties at least up to 1100 °C [11].

In contrast, many conventional oxide-based piezoelectric and especially ferroelectric thin film materials such as Pb_1−x_Zr_x_TiO_3_ or BaTiO_3_ are limited in their usable temperature range either by phase transitions (relatively low Curie temperatures), which describe the transition from the pyroelectric and thus ferroelectric state to a paraelectric state, or chemical decomposition [12,13]. Numerous detailed studies on temperature-dependent physical properties, e.g., the change in coercive fields [14,15], the pyroelectric coefficient [16], thermo-electro-acoustic coupling and thermal expansion [17,18,19], have been conducted to date, but only moderate temperatures of max. 400 °C were investigated. Recently, we also reported on in situ and ex situ high-temperature investigations, addressing the structural stability of Al_1−x_Sc_x_N (x~0.3) thin films [11,20]. However, depending on the annealing conditions, thin film stack and deposition conditions, both degradation of crystal quality and improvement after annealing were observed. Thereafter, the overall picture of the thermal stability of AlScN thin films in the high-temperature regime remains diffuse and a discussion of the influence of the Sc content is still missing. 

Structurally, the substitution of Al atoms by Sc atoms into the AlN_4_ tetrahedra distorts the (Al, Sc)-N bond lengths and bond angles of the three bonds forming the basal plane of the new tetrahedral *M*N_4_ (*M* = Al, Sc) unit. As a result, the in-plane lattice parameter *a* and out-of-plane lattice parameter *c* change anisotropically with increasing concentration of Sc [19]. Statistical calculations on supercell models support this relationship showing that Sc increases disorder and symmetry breaking, establishing a tilt of the *M*N_4_ tetrahedra [21]. In consequence, a pronounced elastic softening along the *c*-axis is observed [17] which couples to the evolution of the internal displacement parameter *u* of the cation–anion distance described by the product *uc*. The compromise between the ideal tetrahedral coordination of Al (*u* = 3/8) and the favored octahedral coordination of Sc (*u* = 1/2) results in the displacement of Sc in the tetrahedra. With increasing Sc concentration, this displacement approaches a theoretical layered hexagonal phase with *u = 0.5* promoting phase transition [21]. The flattened energy landscape with increasing Sc concentration [22] also improves the materials’ piezoelectric response up to *d*_33_~27 pC/N at *x* = 0.43 [23] and promotes ferroelectric switching in high electric fields [1]. In conclusion, the substitution of Al by Sc in AlN in tetrahedral positions destabilizes the wurtzite-type crystal structure, resulting in a phase transformation above *x* > 0.46 to a non-polar cubic AlScN exhibiting rocksalt-type structure with octahedral coordination [24,25].

With this contribution, we extend our previous work on Al_0.73_Sc_0.27_N thin films on templates of Mo(110)/AlN(0001)/Si(001) [11] and match our new results to high-temperature data of Al_1−x_Sc_x_N(0001)/Al_2_O_3_(0001) to provide a joint perspective on AlScN films subjected to the high-temperature regime. In the latter systems, the Sc content was varied from Sc *x* = 0.0 to 0.40 to study Sc content-dependent thermal expansion behavior and high-temperature stability. The discussed results are based on the combination of X-ray diffraction techniques and in situ annealing experiments. Our measurements reveal the increasing structural destabilization of AlScN with increasing Sc content. Further, the demonstration of thermally activated effects is described, which drive an unexpected volume expansion at intermediate and high temperatures exceeding 550 °C. The high-temperature branch of thermal expansion is separated into intrinsic and extrinsic contributions related to oxygen impurities resulting in an irreversible change in the lattice parameters after annealing. Our results could have implications for the integration of AlScN thin films into metal–ferroelectric–metal capacitors for actuator, sensor or computing structures designed for high-temperature operation exceeding 500 °C up to the materials’ Curie temperature of >1100 °C.

## 2. Materials and Methods

### 2.1. Thin Film Samples

Three different sets of Al_1−x_Sc_x_N thin film samples summarized in Table 1 were observed:

(#1) Thin films of Al_0.73_Sc_0.27_N (thickness 400 nm) were deposited on high-temperature-stable metal electrode Mo(110)/AlN(0001)/Si(001) templates using an Oerlikon MSQ200 multi-source pulsed-DC sputter chamber and previously reported processes [26,27]. These films show a typical highly *c*-axis-oriented fiber texture (the 0002 XRD rocking curve (XRC) full-width at half-maxima (FWHM) is ~1.6°) with a small number of misoriented grains [26]. Surface capping of the films with a 100 nm layer of amorphous SiN_x_ was applied to protect the film from oxidation during high-temperature treatment.

(#2) Epitaxial 1 µm thick films of Al_1−x_Sc_x_N (x = 0, 0.09, 0.23, 0.32, 0.40) with *c*-axis-oriented columnar growth were deposited onto sapphire Al_2_O_3_(0001) wafer substrates (diameter: 100 × 100 mm) by reactive pulsed-DC magnetron sputter epitaxy (MSE). For the depositions, an Evatec cluster sputter tool with a base pressure of ~5 × 10^−6^ Pa in a co-sputtering configuration of 99.9995% pure Al and 99.99% pure Sc targets (diameter: 100 mm) was used running a process described in previous studies [17,28]. The FWHMs of XRC scans for the AlScN 0002-reflection are in the range of 0.9 and 1.6° for *x* = 0 and *x* = 0.40, respectively.

(#3) Epitaxial thin films of Al_0.6_Sc_0.4_N (thickness of 400 nm) with *c*-axis-oriented columnar growth on a template of Mo(110)/AlN(0001)/Al_2_O_3_(0001). The films were deposited according to the procedure described in [29] and capped with a 100 nm thick layer of SiN_x_ for passivation during annealing experiments.

### 2.2. X-ray Diffraction Experiments

The temperature-dependent microstructural changes in all thin film samples were investigated via XRD experiments on as-deposited samples as well as during and after in situ experiments. The structural properties of the as-grown epitaxial films (#2) were analyzed on a PANalytical X’Pert Pro MRD diffractometer equipped with a multi-layer mirror and a 2 × Ge 220 monochromator, providing Cu-Kα_1_ radiation (λ = 154.06 pm). XRD experiments on the sample sets #1 and #3 were conducted on a Rigaku SmartLab diffractometer operated under similar conditions. To characterize the as-deposited and annealed samples, phase analyses and mosaic tilt analyses were performed by recording 2θ/θ-scans and rocking curve ω-scans of the AlScN 0002-reflection.

To study effects of high-temperature annealing in (#2) Al_1−x_Sc_x_N/Al_2_O_3_ films as a function of Sc concentration, we performed in situ XRD measurements in the temperature range of 30–1000 °C. The experiments were conducted at a base pressure of ~6 × 10^−3^ mbar achieved inside a graphite dome heating furnace (Anton Paar, Ostfildern-Scharnhausen, GermanyDHS 1100 Domed Hot Stage) placed in a PANalytical Empyrean diffractometer equipped with a PIXcel hybrid detector (Malvern Panbalytical, Kassel, Germany). 2θ/θ and ω/2θ diffraction scans were recorded after 20 min hold time at selected temperatures for approximately 60 min of dwell time. The heating rate was set to 150 °C/min. Lattice parameters were estimated from scans of the symmetrical 0004-reflection and asymmetric 10–15-reflection with shallow (−) and steep (+) angles of incidence ω, similar to the technique described by Herres et al. [30]. For the analysis of sample set #1 and #3, simple 2θ/θ-scans were conducted during in situ heating experiments under similar conditions using an AntonPaar (DHS 1100) hot stage placed on the Rigaku diffractometer and as described in [11].

In addition to the in situ examinations, we also conducted an ex situ annealing experiment to compare the high-temperature behavior of samples exposed to and protected from oxygen-contaminated environments during high-temperature annealing. Here, a Al_0.73_Sc_0.27_N/Mo(110)/AlN(0001)/Si(001) sample was placed inside a self-built tube furnace keeping a quartz tube under vacuum (3–4 × 10^−7^ mbar). Initially, the quartz tube was annealed without the sample to 800 °C together with Ti getter material to reduce the amount of adsorbed oxygen on the tube walls. The actual annealing experiment was started at a base pressure of 1.2 × 10^−6^ mbar and the temperature was increased to 800 °C in steps of 150 °C and cooled down to room temperature in a single step.

## 3. Results

The Results section is structured in two parts. In this first part, in situ annealing experiments on the fiber textured Al_0.73_Sc_0.27_N/Mo(110)/AlN(0001)/Si(001) (sample set #1) thin films are discussed with respect to intrinsic and extrinsic effects of observed anomalous thermal expansion. In the second part, the effect size of the thermal expansion behavior and the microstructural changes to epitaxial thin film samples of Al_1−x_Sc*_x_*N/Al_2_O_3_(0001) are analyzed depending on Sc concentration (0 < *x* < 0.40) (sample set #2). Structural data recorded before and after annealing are discussed and data recorded during annealing will be compared. Eventually, we conclude on our hypotheses considering data from sample #3.

### 3.1. Part A: Intrinsic and Extrinsic Effects of Anomalous Thermal Expansion in AlScN Thin Films

**Figure 1:**
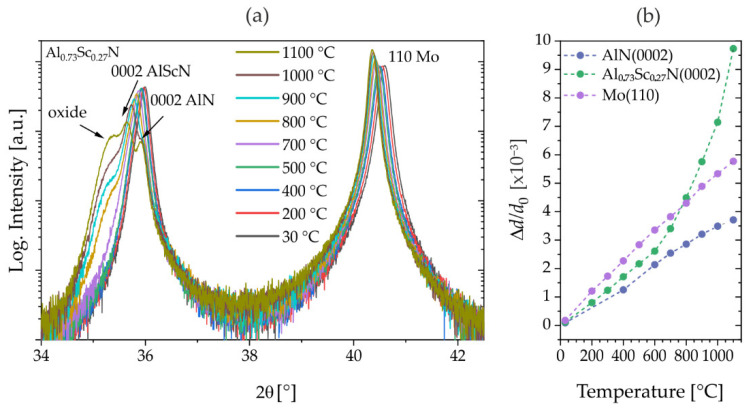
XRD in situ (with oxygen atmosphere) annealing experiments on Al_0.73_Sc_0.27_N and AlN thin films grown on Mo(110)/AlN(0001)/Si(001) (sample set #1). (**a**) 2θ/θ-scan: Evolution of 0002-reflection profile of Al_0.73_Sc_0.27_N and (**b**) relative changes in lattice spacings *d* with temperature calculated from AlN(0002) and Al_0.73_Sc_0.27_N(0002)/Mo(110)-reflections.

The exceptional robustness of the wurtzite-type structure and its ferroelectric polarization switching have been recently demonstrated for 400 nm Al_0.73_Sc_0.27_N/Mo(110)/AlN(0001)/Si(001) thin films during and after high-temperature treatment up to 1100 °C [11]. However, the experimental data suggested a minor degradation of the crystalline quality during the process, which is illustrated by highly comparable types of data collected on Al_0.73_Sc_0.27_N thin films from the identical wafer as presented in Figure 1. The evolution with temperature of the Al_0.73_Sc_0.27_N 0002-reflection profile centered at 2θ~36° shown in Figure 1a displays the decrease in the maximum diffracted intensity of the Al_0.73_Sc_0.27_N component and the development of a hump as a shoulder at lower diffraction angles of the 0002-reflection. These observations indicate the reduction in the sizes of coherently scattering domains of the Al_0.73_Sc_0.27_N phase and the formation of a top oxide layer of (Al,Sc)(N,O)_x_, which is supported by energy-dispersive X-ray spectroscopy measurements on a scanning electron microscope before and after annealing (not shown). No further peaks indicating oxide formation were observed in the temperature-dependent 2θ/θ-scans (30–100 °C) (not shown). Further, the temperature dependent 0002-reflection profiles in Figure 1a reveal the 0002-reflection of the AlN seed layer at a temperature of 1000 °C (see arrows) at higher diffraction angle with respect to the intensity maximum. This could be explained by differences in thermal expansion coefficients of AlN and AlScN, which are known for temperatures up to 400 °C [17]. However, when treating AlScN films at high temperatures, a transition in thermal expansion is observed. The comparisons between the thermal shifts in the 0002-reflection maxima for Al_0.73_Sc_0.27_N and AlN thin films and the Mo(110)-reflection of the Mo layer underneath Al_0.73_Sc_0.27_N are presented in Figure 1b. Here, the relative change in the lattice spacings Δ*d[T]*/*d*_0_ reveals strong non-linearity in the thermal lattice expansion of the Al_0.73_Sc_0.27_N film. The curve for the Al_0.73_Sc_0.27_N film (green curve in Figure 1b) shows a non-linearity in thermal expansion changing from a linear low-temperature branch to a high-temperature branch at a transition temperature of about *T_tr_*~600 °C. The linear slope of the low-temperature branch is (Δ*c*[25 °C–600 °C]/*c*_0_)/Δ*T*~4.4 × 10^−6^/°C and increases by a factor of 3 to (Δ*c*[700 °C–1000 °C]/*c*_0_)/Δ*T*~13.7 × 10^−6^/°C in linear approximation to the high-temperature branch. Any influence of the underlayer is excluded by comparison to the relative change in the underlayer’s Mo(110)-reflection which shows highly linear expansion. Further, an AlN film deposited under identical conditions was heated and shows a linear expansion of (Δ*c*[25 °C–1100 °C]/*c*_0_)/Δ*T*~3.5 × 10^−6^/°C. Due to the rather rough linear approximation over the entire temperature interval, the value for AlN is somewhat smaller but still consistent with literature data of 4.2 [20–800 °C] − 4.65 [20–400 °C] × 10^−6^/°C [17,31].

To our knowledge, this strongly non-linear transition has not been observed to date for AlScN thin films. In the case of many oxide materials, non-linear thermal expansion behavior can be discussed in the context of oxygen- or oxygen vacancy-related effects termed ‘chemical expansion’ [32]. These chemical expansion phenomena can be non-reversible or reversible in nature. For instance, non-reversibility is observed in the case of cation–oxygen networks forming in metallic glasses [33] or small structural transitions in (Ba_0.5_Sr_0.5_)TiO_3_ induced by a change in oxygen site occupancy [34]. Reversible phenomena are commonly observed in non-stoichiometric perovskite-based ion-conducting ceramics which show pronounced chemical expansion depending on the oxygen partial pressure and temperature [35,36].

Parallel to the strong oxidation of the AlScN film, the interaction with oxygen species and the intrinsic defect structure of the material could lead to the strong thermal expansion and irreversible changes. In this respect, the fiber textured columnar films could potentially provide a Sc- and oxygen-enriched grain boundary structure [37], which could promote pathways for atmospheric oxygen species into the material. 

**Figure 2:**
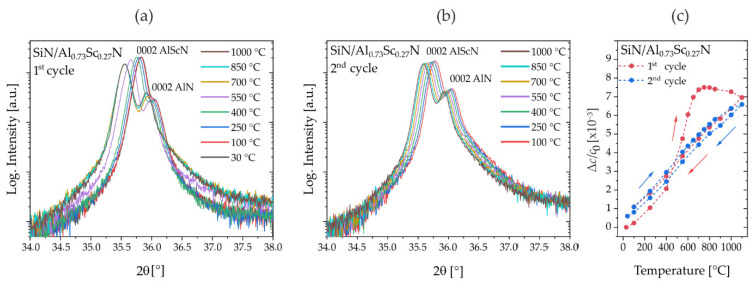
XRD in situ (with oxygen atmosphere) annealing experiments. Observation of anomalous intrinsic lattice expansion on oxygen-protected SiN passivated thin films of Al_0.73_Sc_0.27_N(0001)/Mo(110)/AlN(0001)/Si(100) (sample set #1). (**a**) 2θ/θ-scan: Evolution of the 0002-reflection profile during the first cycle. (**b**) 2θ/θ-scan: Evolution of the 0002-reflection profile during the second cycle. (**c**) Relative change Δ*c*/*c*_0_ in lattice parameter with annealing temperature in two consecutive annealing cycles.

These preliminary experiments suggest the presence of residual oxygen contamination of the annealing atmosphere inside the graphitic dome placed in the diffractometer. Hence, the Al_0.73_Sc_0.27_N film was capped with a 100 nm thick SiN_x_ layer to protect the film surface from oxidation-dependent effects, allowing us to investigate the purely intrinsic material contribution to the transition in thermal expansion behavior. With this experimental design, new in situ annealing experiments were conducted up to 1100 °C. The evolution of reflection profiles during the first and second annealing cycle and the corresponding Δ*c(T)/c*_0_ plots are shown in Figure 2**.** The reflection profiles depicted in Figure 2a exhibit neither an oxide hump, nor a strong decrease in the reflection intensity, which is a sign of improved structural stability due to avoiding surface oxidation. Instead, a negligible XRD reflection broadening is observed by the increase in background intensity at higher diffraction angles at 550 °C, which could be due to changes in the average crystallite size, accumulation of defects and local lattice strains. Upon further annealing, no further changes in the reflection profile (Figure 2b) are observed for a second temperature cycle, indicating completed activation of any intrinsic processes until the applied temperature of 1100 °C.

The corresponding Δ*c(T)/c*_0_ plots for the first and second full temperature cycle are displayed in Figure 2c. In the first cycle (red curve), the thermal expansion in the low-temperature regime is consistent with uncapped films with (Δ*c*[25 °C–400 °C]/*c*_0_)/Δ*T*~6.0 × 10^−6^/°C (compare Figure 1b) and literature reference data (6.38 × 10^−6^ K^−1^) for Al_0.68_Sc_0.32_N/Al_2_O_3_ [17]. However, a new and purely intrinsic regime at intermediate temperatures is observed with (Δ*c*[450 °C–650 °C]/*c*_0_)/Δ*T*~22.2 × 10^−6^/°C with much higher expansion and lower transition temperature *T_tr_*~450 °C. This value of thermal expansion is almost double compared to the uncapped film at high temperatures with *T_tr_* > 600 °C. In the high-temperature regime of >650 °C, the expansion slows down and reverses its sign (Δ*c*[750 °C–1100 °C])/*c*_0_)*/*Δ*T*~−1.5 × 10^−6^/°C. After cooling down to room temperature, linear thermal expansion over the entire temperature regime with (Δ*c*[1100 °C–30 °C]/*c*_0_)*/*Δ*T*~ −6.0 × 10^−6^/°C is observed and a small irreversible change in the *c* lattice parameter remains at Δ*c/c*_0_~0.5 × 10^−3^, which is much smaller than for the uncapped film. In a second temperature cycle (blue curve in Figure 2c), no anomalous thermal expansion is observed, consistent with the reflection profiles. 

The comparison of both experiments suggests that oxidation effects or the interaction of material defects with oxygen species play a prominent role for high-temperature lattice expansion at >800 °C and the irreversible change in respective lattice parameters at room temperature.

This hypothesis is supported by the comparison of in situ XRD experiments with oxygen in the annealing atmosphere and ex situ XRD experiments without available oxygen in the atmosphere performed on uncapped films of Al_0.73_Sc_0.27_N/Mo(110)/AlN(0001)/Si(001). In this study, the first sample was introduced into the in situ XRD analysis when performing two consecutive heating cycles. The second sample was placed into a quartz tube furnace which was evacuated to 10^−7^ mbar. Both samples were treated with identical temperature profiles. In the first heating cycles, a maximum temperature of 800 °C was applied, which is about the temperature at which the positive slope of the intrinsic expansion reverses, whereas a maximum of 1000 °C was used in the second in situ heating hysteresis. After the first ex situ cycle, a second temperature cycle was performed in situ to evaluate the 2θ shifts in the 0002-reflection at higher temperatures.

**Figure 3:**
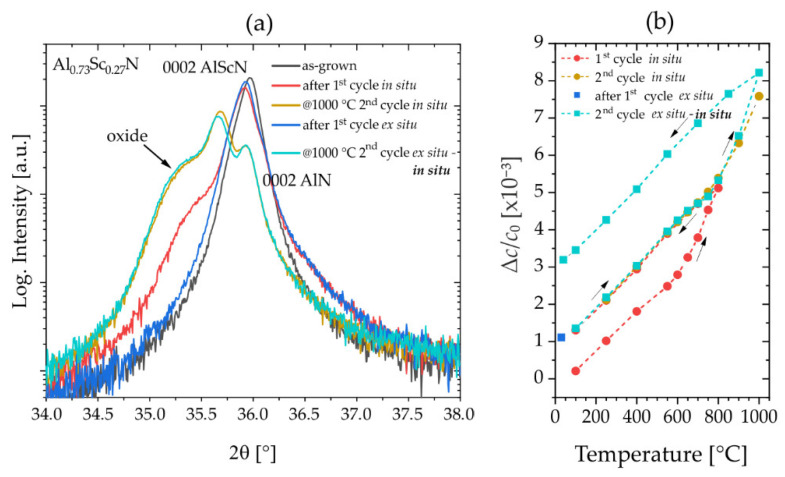
XRD annealing experiments on Al_0.73_Sc_0.27_N thin films grown on Mo(110)/AlN(0001)/Si(001) (sample set #1). (**a**) 2θ/θ-scans: Evolution of 0002-reflection profile after ex situ (without oxygen atmosphere) and during in situ (with oxygen atmosphere) experiments. (**b**) Relative changes in lattice parameter *c* for Al_0.73_Sc_0.27_N thin films annealed ex situ and in situ.

The reflection profiles and plots of the relative thermal expansion are summarized in Figure 3. Figure 3a shows the respective reflection profiles at selected stages during the experiment. As expected, no oxide hump is observed after the ex situ annealing (blue line), suggesting a purely intrinsic thermal expansion behavior. For the in situ annealed sample (red line), the oxide hump is observed. However, after performing the first cycles, both samples show an irreversible change in lattice parameter of about *Δc/c*_0_~1 × 10^−3^ in the plots of the relative thermal expansion shown in Figure 3b. This could indicate that the observed oxidation does not have a major effect on the expansion at intermediate temperatures and that the intrinsic contribution is dominating. By stopping the first cycle at 800 °C, it is assumed that the intrinsic effects on the anomalously high positive thermal expansion have all been activated and no further reaction would be observed. Indeed, this holds true for both samples in the second cycle up to 800 °C (golden and turquoise lines). By passing the 800 °C mark, comparable thermal expansion is observed when residual oxygen is supplied by the annealing atmosphere which results in a strong oxidation and large irreversible lattice changes *Δc/c*_0_~3 × 10^−3^ after annealing.

In summary, anomalous high thermal expansion and related irreversible lattice changes have been observed upon thermal activation by annealing of AlScN thin films. The experiments suggest an intrinsic material specific contribution activated at intermediate temperatures of >600–800 °C which is superimposed with extrinsic effects acting in parallel to oxidation of the films at temperatures exceeding 800 °C, if not protected by a surface covering layer. 

### 3.2. Part B: Discussion of Scandium Concentration in Al_1−x_Sc_x_N(0001)/Al_2_O_3_(0001) Thin Films

**Figure 4:**
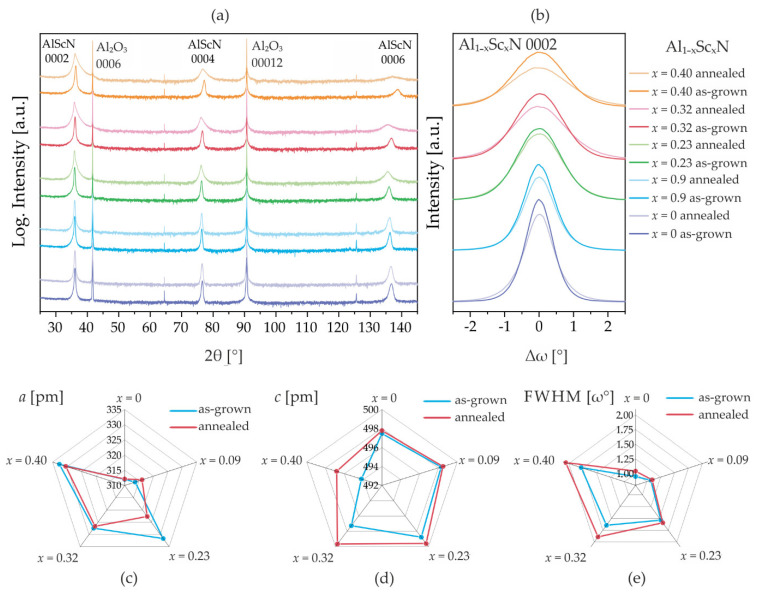
Analysis of Al_1−x_Sc_x_N/Al_2_O_3_ (0 ≤ x ≤ 40.0) before and after in situ thermal annealing (sample set #2). (**a**) XRD 2θ/θ diffractograms. (**b**) XRD rocking curve measurements of the 0002-reflections. Summary of changes in lattice parameters *a* (**c**) and *c* (**d**) calculated from symmetric and asymmetric measurements, ω-FWHM (**e**) calculated from the XRD rocking curves.

In this section, the discussion is turned towards epitaxial *c*-axis columnar grown thin films of 1 µm Al_1−x_Sc*_x_*N on Al_2_O_3_ (sample set #2). The recorded XRD 2θ/θ-scans for 1 μm thick Al_1−x_Sc*_x_*N (0 < *x* < 0.40) films before and after thermal annealing are shown in Figure 4a. All diffractograms of the as-grown sputtered Al_1−x_Sc*_x_*N thin films demonstrate exclusive *c*-axis orientation as well as fixed in-plane orientation described by epitaxial relationships (0001)AlScN//(0001)Al_2_O_3_ and (10–10)lScN//(11–20)Al_2_O_3_, respectively [17]. After annealing in situ (with oxygen atmosphere) to 1000 °C, only the 000*ℓ* (*ℓ*  =  2, 4, 6)-reflections are observed, indicating decent temperature stability of the wurtzite-type phase for all examined Sc concentrations *x*. 

However, a considerable XRD reflection broadening of the 000*ℓ*-reflection profiles is observed after annealing for Sc concentrations x ≥ 0.23. This is paired with a shift in the reflection position to lower 2θ values. The broadening of the 000*ℓ*-reflections features asymmetry, with the right tail of the Bragg reflection becoming extended relative to the left tail. No hump on the 0002-reflections is observed, which could indicate a better stability against oxidation for the epitaxial films with fewer grain boundaries. Indeed, rocking curve measurements of the 0002-reflection (Figure 4b) confirm this broadening and provide evidence for a structural degradation by the reduction in maximum diffracted intensity.

The XRD reflection broadening is mainly attributed to reduced sizes of coherently scattering domains, the accumulation of defects and local lattice strain. A shift in the diffraction angle to lower values of 2θ in consequence of annealing is related to the expansion of the *c* lattice parameter, as discussed in part A. However, the exact origin of the reflection broadening remains speculative without a structure model and Rietveld refinement. Instrumental broadening of the reflections can be neglected due to the high mosaicity of the AlScN films. A detailed analysis of the in-plane and out-of-plane lattice parameter changes using data from the symmetric 2θ/θ-scans and asymmetric ω/2θ 10–15(−) and 10–15(+)-reflection scans [30] is performed and the results are summarized in Figure 4c and Table 2. From the comparison, it is apparent that the high-temperature annealing induces irreversible changes to the lattice parameters of Al_1−x_Sc*_x_*N. These changes manifest in the reduction in the *a* parameter and the increase in the *c* parameter in combination with the degradation of the overall crystal quality (broadening of FWHM). A clear trend is visible in the magnitude of the effect which seems to scale with Sc concentration *x* indicating an increasing instability of high-Sc alloys at elevated temperatures. For instance, in the case of low-Sc content Al_0.91_Sc_0.09_N films, the irreversible change in the *c* lattice parameter is Δ*c/c*_0_ 0.04% in contrast to high-Sc content Al_0.60_Sc_0.40_N films showing Δ*c/c*_0_~0.5%, associated with a relative broadening of FWHM of 2.2% and 15.7%, respectively. We note that the film with x = 0.23 shows a very large unexpected change in the *a* parameter, but follows the general trend regarding the other parameters.

The structural origin of the observed reflection broadening in high-Sc content films could also be related to the competition between the hexagonal wurtzite-type phase and the cubic rocksalt-type structure when approaching 46% Sc [23,24,25,38]. High-temperature annealing could result in local phase destabilization and formation of nanosized cubic domains in Sc-enriched regions, e.g., at defect sites or grain boundaries [37]. Such nanosized domains, as well as the migration of defects, e.g., dislocations, will lead to reflection broadening and asymmetry by diffuse scattering and the formation of low-intensity shoulders in diffraction patterns [39,40]. Typically, we would expect a phase transition to be reversible, but here the strong and non-reversible increase in lattice parameters seems to indicate irreversibility.

**Figure 5:**
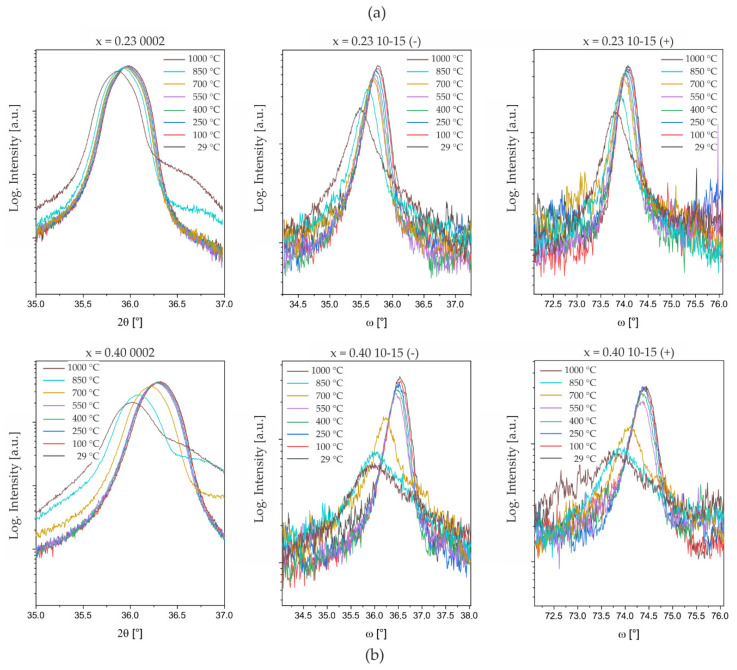
Temperature-dependent changes in symmetric 2θ/θ 0002- and asymmetric ω/2θ 10–15-reflection profiles for (**a**) Al_0.77_Sc_0.23_N and (**b**) Al_0.60_Sc_0.40_N compositions (sample set #2). 10–15-reflections are measured with shallow (−) and steep (+) angles of incidence ω, see [30] for more information on the method.

In further analysis of the in situ annealing experiments, the temperature-dependent changes in the reflection profiles are followed individually. In Figure 5, thin films of Al_0.77_Sc_0.23_N and Al_0.60_Sc_0.40_N are compared to demonstrate the difference between intermediate and high Sc contents. When comparing the reflection profiles, it is directly apparent that the Sc content influences the structural stability and the activation temperature of the observed degradation effects. The evolution of both the symmetric 2θ/θ 0002- and asymmetric ω/2θ 10–15-reflection profiles shows a strong degradation of the initial crystalline quality. The loss of structural coherence is most pronounced in the low-intensity asymmetric reflections, which limits the precise calculation of in-plane parameters at higher temperatures and after annealing. Concerning the XRD reflection broadening, for Sc *x* = 0.23, the elevation of the background tails starts at a temperature of 850 °C, whereas for Sc *x* = 0.40 these features are already observed at 700 °C and with larger magnitude (Figure 5). 

**Figure 6:**
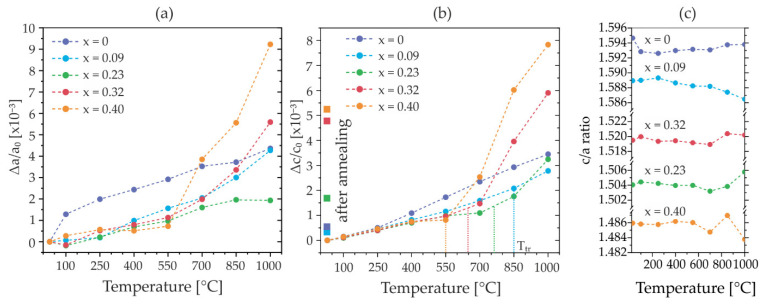
XRD in situ (with oxygen atmosphere) characterization of thermal evolution of the lattice parameters (sample set #2). (**a**) Relative change in the lattice parameter *a* and (**b**) lattice parameter *c* indicating a high-temperature regime of isotropic thermal expansion and estimated values of the transition temperature *T_tr_*. Dashed lines serve as a guide to the eye only. Residual values of lattice expansion after annealing are shown as square data points as given in Table 2. (**c**) Calculated *c*/*a* ratios showing approximately isotropic thermal expansion for the investigated thin films across the full temperature range.

As before, the temperature-dependent changes in the reflection positions are used to calculate the relative thermal expansion of both *c* and *a* lattice parameters. The resulting thermal expansion is illustrated in Figure 6. Here, the relative changes in the *c* and *a* lattice parameters over the temperature range 25–1000 °C are presented for the investigated films in Figure 6a,b, respectively. In this analysis, the transition between the two expansion regimes is evidenced for all Al_1−x_Sc*_x_*N compositions but not for AlN. Note that the films are not protected against available oxygen species, hence, intrinsic and extrinsic processes adding to the thermal expansion are superimposed. The transition from almost linear and low-value expansion at moderate temperatures into large values of expansion at high temperatures is observed for *a* and *c*. This expansion of the lattice is isotropic which is visible from the almost constant *c/a* ratio plotted in Figure 6c. The transition temperature *T_tr_* seems to be related to the Sc content of the film, as well as the magnitude of the expansion. Roughly estimated values from the plots are *T_tr_*~850 °C for Al_0.91_Sc_0.09_N and *T_tr_*~550 °C for Al_0.60_Sc_0.40_N films as indicated by the vertical dotted lines in Figure 6b. In detail, the thermal expansion behavior in the low-temperature regime (<550 °C) is highly comparable for all Sc concentrations, in agreement with previous studies within this temperature range [17]. In strong contrast, the high-temperature regime is characterized by a manifold increase in the expansion, depending on the Sc concentration (up to ~8-fold for Al_0.60_Sc_0.40_N). After cooling back to room temperature, the lattice parameters show irreversible changes as discussed for Figure 4 and Table 2.

**Figure 7:**
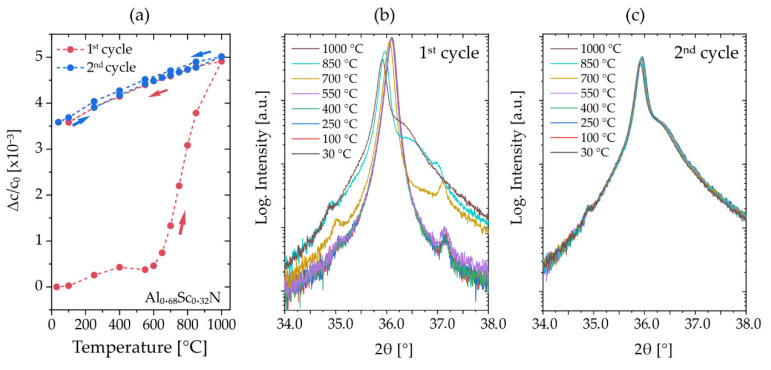
(**a**) XRD in situ (with oxygen atmosphere) temperature cycles in the example of Al_0.68_Sc_0.32_N (sample set #2). (**b**) Evolution of 0002-reflection profile in the first annealing cycle. (**c**) 2θ/θ-scan: Evolution of 0002-reflection profile in the second annealing cycle.

For direct comparison with fiber textured samples from set #1, XRD measurements during two temperature cycles were conducted for the Al_0.68_Sc_0.32_N film, Figure 7. The displayed temperature cycle and reflection profiles are highly congruent to the data recorded on the fiber textured thin films with similar composition. An irreversible change of Δ*c*/*c*_0_~3.5 × 10^−3^ remains after annealing at 1000 °C (Figure 7a) and no further changes are observed in the second temperature hysteresis, congruent with the measured reflection profiles shown in Figure 7b,c. 

## 4. Discussion

The described experiments reveal high-temperature effects related to structural degradation and irreversible anomalous non-linear thermal expansion behavior in AlScN thin films depending on Sc content and film microstructure. The non-linear thermal expansion is described by a transition from an initial expansion regime to a fast-expanding high-temperature regime which is divided into intrinsic and extrinsic contributions, dependent on the availability of oxygen. Available oxygen is believed to lead to the oxidation of the material dependent on the film microstructure (oxidation was only observed for fiber textured films with higher density of grain boundaries) but also enhances the expansion effect size at *T* > 800 °C and stabilizes the increased lattice parameters. The effect size of high-temperature expansion scales with the Sc content of the films, which could be related to a higher defect density leading to the increased destabilization and easier oxidation. A further indicator for the scaling defect density is the increasing magnitude of the peak broadening, which was discussed as an indicator for defect movement as well. Besides dislocations or grain boundaries, point defects such as nitrogen vacancies V_N_ are one major type of defect in AlN and AlScN thin films. Previous studies by Harris et al. have demonstrated that the wurtzite-type structure of AlN can incorporate up to 0.75 at% oxygen under thermal equilibrium conditions by substitution of N (O_N_) associated with the formation of Al vacancies (V_Al_) [41]. Further insights into the structure relation of AlN films and oxygen are provided by DFT modeling by Gasparotto et al. [42] showing that oxygen can have significant influence on the lattice parameters.

The discussed examples show potential interrelations of oxygen-induced defect structures and changes to the lattice parameters. As observed in this work on AlScN thin films, such phenomena could provide valid explanation for the discussed intrinsic and extrinsic contributions to the anomalous thermal expansion at high temperatures. In addition, Sc has strong affinity for oxygen [43,44,45], and the intrinsic oxygen contamination of AlScN thin films is supposed to scale with the Sc content as well. In this respect, Sc- and oxygen-enriched grain boundaries were already evidenced [37] and the photoluminescence emission of low-Sc films (x = 0.05) was already revealed to be dominated by oxygen defects [46]. Further, modeling of the point defects in rocksalt AlScN resulted in preferable defect complexes of substitutional and interstitial oxygen (O_N_ + O_i_) [47]. The observed oxygen content-dependent destabilization of the lattice in Al-O-N is also consistent with the increasing degradation of the crystal quality with Sc concentration and the formation of oxide phases. 

**Figure 8:**
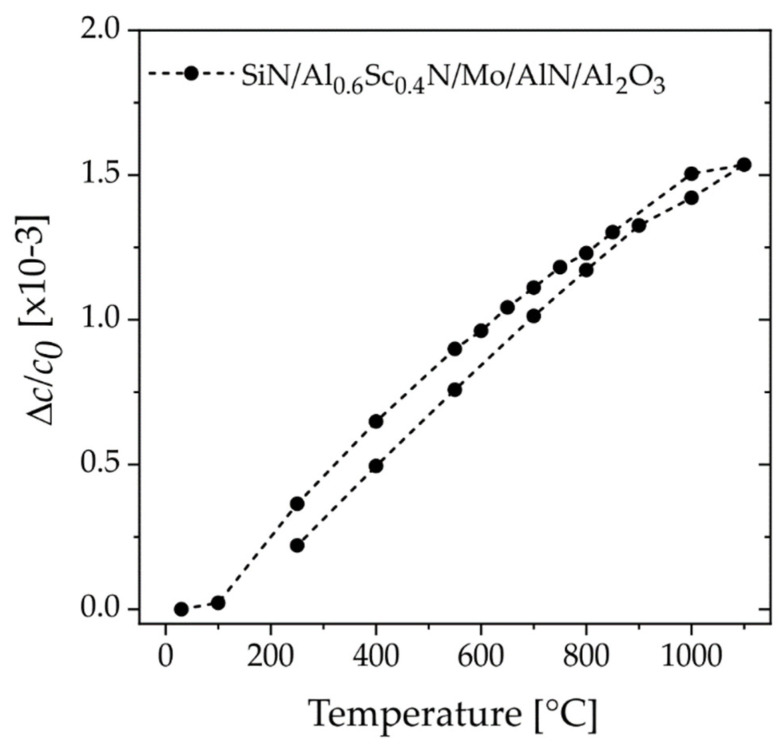
*XRD* in situ (with oxygen atmosphere) experiment. Relative change in lattice parameter *c* with temperature for epitaxial SiN_x_/Al_0.6_Sc_0.4_N(0001) on a template of epi-Mo(110)/AlN(0001)/Al_2_O_3_(0001) (sample set #3).

To support the above discussion, we designed a third sample of a SiN_x_-capped AlScN film with high concentration of Sc *x* = 0.4 grown epitaxially on a sapphire substrate, i.e., SiN_x_/Al_0.6_Sc_0.4_N(0001)/epi-Mo(110)/AlN(0001)/Al_2_O_3_(0001). For such systems, previous work [29] demonstrated that the special epi-Mo(110) electrode provides a growth template for AlScN with one-dimensional single crystalline properties and low defect density. The following assumptions are made: First, the SiN barrier should protect the thin film from oxidation and interaction of oxygen species with the internal defect structure at high temperatures. Second, by choosing a high amount of Sc, any effect magnitude is expected to be large. Third, this AlScN thin film exhibits high crystalline quality with an FWHM~0.7° (from XRC) indicating highly oriented columnar grains originating from epitaxial growth and a low defect density. Hence, the effect of any intrinsic, defect-driven anomalous expansion should be strongly limited. Indeed, the recorded thermal expansion during the first temperature cycle shown in Figure 8 shows almost linear expansion featuring no anomalous behavior. This observation, although not expected so clearly, provides supporting evidence for the above hypothesis.

In summary, the irreversible changes in the lattice parameters of Al_1−x_Sc_x_N/Al_2_O_3_ when exposed to atmospheric oxygen present in low-vacuum conditions are comparable to fiber textured systems. The estimated transition temperatures between the low-temperature expansion and high-temperature expansion regimes and the magnitude of the superimposed intrinsic and extrinsic contributions show a clear trend with the Sc content of the films. With increasing Sc content, the transition temperature is decreased and the effect sizes of thermal expansion and degradation of crystallinity are increased. The anomalous thermal expansion can only be activated by temperature once. Further, the microstructure and possibly the defect structure seem to impact the stability to oxidation of the films, which seems not to be interrelated with the extrinsic part of high-temperature thermal expansion. However, at very high temperatures >800 °C, hypothetically, oxygen species could diffuse into the material via defects and grain boundaries and interact with thermally activated defect sites to further drive the lattice expansion and act as obstacles to stabilize the expanded lattice. Scandium is known to destabilize the AlN lattice by its larger cation size and structural preference for octahedral coordination. Hence, increasing its concentration in AlN could induce higher defect densities, which are seemingly possible to activate at lower temperatures by the flattened energy landscape.

## 5. Conclusions

The thermal stability and temperature-induced effects of AlScN thin film samples with different microstructures based on the growth template were investigated by in situ XRD. The degradation of the crystalline quality and a remnant lattice expansion in the *c*-direction of up to 0.5% for epitaxial Al_0.60_Sc_0.40_N(0001)/Al_2_O_3_(0001) were observed as a function of Sc concentration. There is first evidence that the remnant expansion is related to the activation of intrinsic defects and the films’ oxygen affinity at elevated temperatures, which are accounted for as intrinsic and extrinsic sources of anomalous thermal expansion. The understanding of the exact details of these phenomena provides opportunity for further investigations on the exact type of defect structures using more advanced methods, such as positron annihilation spectroscopy [48,49,50]. The detailed understanding of the intrinsic defect structures and temperature activation effects is of high importance for the integration of sputtered AlScN layers into sandwich structures, e.g., for ferroelectric field-effect transistor memory capacitors or ferroelectric tunnel junctions. That is especially relevant with respect to downscaling of the layer thickness and high-temperature operation, where oxidation of the functional layer has to be prohibited and an anomalously high expansion of the AlScN crystal lattice puts increased stresses on any neighboring crystalline layers to avoid device failure via crack formation or delamination. In conclusion, our investigation emphasizes the requirement of a low density of material defects in AlScN thin films when operating at high temperatures and the benefit of integration with a protecting top layer, e.g., a temperature-resistant electrode such as Mo or NbN. 

## Figures and Tables

**Table 1:** Summary of all thin film samples investigated in this study.

Al_1−x_Sc_x_N(0001)	#1	#2	#3
Template	Mo(110)/AlN(0001)/Si(001)	Al_2_O_3_(0001)	Mo(110)/AlN(0001)/Al_2_O_3_(0001)
Sc concentration *x*	0.27	0, 0.09, 0.23, 0.32, 0.40	0.40
Thickness	400 nm	1000 nm	400 nm
Microstructure	Fiber textured	Epitaxial with columnar growth	Epitaxial with columnar growth
XRC (FWHM)	1.6°	0.9–1.6°	0.7
SiN_x_ passivation	As specified	No	Yes

**Table 2:** Lattice parameters and mosaicity analyses of Al_1−x_Sc_x_N (0 ≤ x ≤ 0.4) thin films by XRD before and after thermal annealing (sample set #2).

	a [pm]			c [pm]			ω-FWHM [°]		
Sample	As-Grown	Annealed	Δa/a_0_ [×10^−3^]	As-Grown	Annealed	Δc/c_0_ [×10^−3^]	As-Grown	Annealed	Δω/ω_0_ [×10^−2^]
AlN	312.0	312.1	0.468	497.5	497.8	0.64	0.95	1.04	9.8
Al_0.91_Sc_0.09_N	313.6	316.0	7.64	498.3	498.5	0.39	1.07	1.10	2.2
Al_0.77_Sc_0.23_N	331.6	322.6	−27.13	498.8	499.6	1.66	1.53	1.59	3.7
Al_0.68_Sc_0.32_N	327.4	326.7	−2.38	497.3	499.7	4.80	1.64	1.89	14.9
Al_0.60_Sc_0.40_N	332.7	330.5	−6.61	494.2	496.8	5.33	1.78	2.06	15.7

## Data Availability

Original XRD data are available from the corresponding authors upon reasonable request.
